# Effect of Medication Management at Home via Pharmacist-Led Home Televisits: Protocol for a Cluster Randomized Controlled Trial

**DOI:** 10.2196/65141

**Published:** 2025-02-05

**Authors:** Sheikh Rubana Hossain, Akanksha N Samant, Briana C Balsamo, Chelsea E Hawley, Michael C Zanchelli, Carolyn Zhu, Maria D Venegas, Marina Robertson, Megan B McCullough, Judith L Beizer, Kenneth S Boockvar, Albert L Siu, Lauren R Moo, William W Hung

**Affiliations:** 1 James J Peters VA Medical Center Bronx, NY United States; 2 Pharmacy Department James J Peters VA Medical Center Bronx, NY United States; 3 New England Geriatric Research Education and Clinical Center Bedford, MA United States; 4 Department of Medicine Boston University Aram V Chobanian & Edward Avedisian School of Medicine Boston, MA United States; 5 Department of Geriatrics and Palliative Medicine Icahn School of Medicine at Mount Sinai New York, NY United States; 6 Center for Healthcare Organization and Implementation Research Bedford VA Medical Center Bedford, MA United States; 7 Department of Health Policy and Management Boston University School of Public Health Boston, MA United States; 8 College of Pharmacy and Health Sciences St John’s University New York, NY United States; 9 University of Alabama at Birmingham Birmingham, AL United States; 10 Department of Neurology Massachusetts General Hospital and Harvard Medical School Boston, MA United States

**Keywords:** older adults, medication management, televisit, polypharmacy, adverse drug reaction

## Abstract

**Background:**

Older adults are more likely to have multiple chronic conditions, be prescribed multiple medications, and be more susceptible to adverse drug reactions (ADRs) to their medications. In addition, older adults often use over-the-counter medications and supplements, further complicating their medication regimens. Complex medication regimens are potentially harmful to older adults. Interventions aimed at reducing medication discrepancy in the ambulatory clinic setting, such as reviews of medication lists and the implementation of “brown bag” reconciliation, continue to be challenging, with limited success. Pharmacist-led interventions to improve appropriate medication use in older adults have demonstrated effectiveness in reducing ADRs. Video visits have the potential to provide direct visualization of medications in older adults’ homes, thereby reducing medication discrepancy and increasing medication adherence. Pharmacist-led management of older adults’ medication regimens may improve appropriate medication use in older adults.

**Objective:**

The objective of this study is to examine the effect of pharmacist-led medication through home televisits compared to usual care on appropriate medication use, medication discrepancies, medication adherence, and ADRs.

**Methods:**

We will conduct a 2-site cluster randomized controlled trial (RCT). The intervention will be a pharmacist-led home televisit including medication reconciliation and assessment of actual medication use. The cluster RCT was iteratively adapted after a pilot test. The primary outcome of medication appropriateness of the intervention will be measured using the STOPP (Screening Tool of Older Persons’ Prescriptions) criteria for potentially inappropriate medications (PIMs) at 6 months. Medication lists obtained will be compared against electronic medical records (EMRs) by a clinician to establish discrepancies in medications. The clinician will review medications using the validated Medication Appropriateness Index (MAI).

**Results:**

This project has been peer-reviewed and selected for support by the Veterans Affairs (VA) Health Services Research Service. The pilot phase of the study was completed December 2021 with 20 veterans and was primarily informed by the Steinman model of the prescribing process adapted to include system- and provider-level factors. The last date of enrollment was August 6, 2021. We anticipate the completion of the ongoing trial in spring 2025. The first results are expected to be submitted for publication in 2025.

**Conclusions:**

The cluster RCT will provide evidence on medication management through televisits. If found effective in improving the use of medications, the intervention has the potential to impact older adults with multiple chronic conditions and polypharmacy.

**Trial Registration:**

ClinicalTrials.gov NCT04340570; https://clinicaltrials.gov/study/NCT04340570

**International Registered Report Identifier (IRRID):**

PRR1-10.2196/65141

## Introduction

### Polypharmacy in Older Adults and the Importance of Medication Reconciliation

The US population is aging rapidly; the population aged 65 years and above is projected to be doubled by 2050. The most rapidly growing segment is the population of older adults aged 85 years and above, which will more than triple from 5.9 million in 2012 to 18 million in 2050 [1,2]. Older adults aged 65 years and above often have multiple chronic diseases—more than 60% have 2 or more chronic diseases [3], and 17% have 4 or more chronic diseases [4]. Older adults with multiple chronic comorbidities often require multiple medications for management, particularly with guideline-based management of chronic diseases [5,6]. The use of multiple medications in older adults is common, with almost 20% of older adults aged 65 years and above taking 10 or more medications [7,8]. Multiple-medication use in older adults is associated with a lower adherence rate and increased use of inappropriate medications [9,10]. The use of multiple medications increases the risk for potential drug interactions, leading to undesirable adverse drug reactions (ADRs), which could also contribute to lower medication adherence. In particular, older adults are more susceptible to ADRs due to the changes in their physiology, clearance, and reserves [11], particularly with polypharmacy [9,12] Based on the phycological changes in the older population, knowing the drug regimens with older adults is an important feature in order to provide medication safety and make adjustment to their regimen accordingly to ensure appropriate medication use. The START/STOPP (Screening Tool to Alert to Right Treatment/Screening Tool of Older Persons’ Prescriptions) criteria [13-15] and American Geriatrics Society (AGS) Beers criteria [16,17] for potentially inappropriate medications (PIMs) were developed to provide evidence-based guides to signal clinicians about medications that are potentially inappropriate and to enhance medication appropriateness. These medications include those with strong anticholinergic properties, which may disproportionately affect older adults and are linked to adverse outcomes of ADRs, and other medications that are demonstrated to have significant side effects in older adults.

### Use of Telemedicine to Improve Medication Use in Older Adults

Telemedicine is a modern visit option enabled by advances in telecommunications technology [18]. The use of telemedicine in older adults has been examined in prior small studies that have demonstrated feasibility, acceptability, and user satisfaction [19-22]. Extending televisits to patients’ homes has the potential to impact many aspects of care that rely on patient self-management, such as medication use. Although telephone-based pharmacist interventions have the potential to impact medication use at home [23,24], they still rely on accurate information reported by older adults over the phone without a mechanism for confirmation. The addition of video has the potential to further enhance the visit [25,26] by (1) visual ascertainment of actual medications taken by patients, (2) visual demonstration of the patients’ actual use of medications, and (3) education of patients on proper use. Although in-person home-based reconciliation has the potential to improve accurate appraisal of medication use and reconciliation, it is not feasible for wide adoption as it is resource intensive. The study proposed here will examine the impact of home televisits by pharmacists on patients at high risk for ADRs (ie, with polypharmacy and multiple chronic conditions).

## Methods

### Research Design Overview

This study is designed as a mixed methods hybrid type 1 effectiveness implementation study [27], where we will test the health impact of pharmacy televisits, while also collecting data on the implementation process to facilitate subsequent scale-up efforts [28]. As older adults have higher rates of chronic conditions and polypharmacy, we included veterans aged 65 years and above in this study. The inclusion criterion of 5 or more medications is based on prior literature on polypharmacy and findings of increased risk for drug interactions and ADRs. The enrollment criteria are consistent with and exceed the criteria set in fiscal year 2019 by the Center for Medicare Services for provision of medication therapy management in Medicare Part D [29]. Among patient characteristics, polypharmacy (≥5 medications) and multiple chronic conditions are considered important risk factors for ADRs and are highly prevalent among frail older adults at risk for ADRs [30].

The intervention for the cluster randomized controlled trial (RCT) was iteratively adapted after a pilot test with 20 veterans [31]. Home televisits, where pharmacists conducted medication reconciliation and management, were refined, after which a formative evaluation [32] was conducted, guided by the Consolidated Framework for Implementation Research (CFIR) [33]. Data were gathered in several ways: (1) enrolled veterans (n=20) were observed by study staff present within the veterans’ homes during the clinical pharmacist home televisit, and (2) study staff administered a postencounter questionnaire with the goal of improving the veteran-based technical experience and clinical encounter. The data points were used to adapt and make changes to the intervention for the cluster RCT.

The cluster RCT is registered on ClinicalTrials (NCT04340570). The intervention will be a pharmacist-led home televisit including medication reconciliation and assessment of actual medication use. Pharmacists will review medication appropriateness using evidence-based criteria and provide recommendations for change of medication use to the patients’ primary patient-aligned care team (PACT). PACTs provide team-based care and consist of a primary care provider (PCP), a registered nurse, a medical assistant, and, often, either a social worker or a pharmacist. In the control group, participants will receive usual care in which medication reconciliation and review will be conducted in clinics by primary care teams. As a primary outcome, we will examine the effect of the intervention on the number of veterans with PIMs, as determined using evidence-based criteria. As secondary outcomes, we will determine the number of PIMs, medication discrepancy using data from record reviews and interviews, and medication appropriateness using validated instruments at 6 months after the intervention. We will also compare the intervention’s effects on the incidence of ADRs using data from record reviews and interviews. To assess the potential for future implementation of the intervention, we will administer postintervention questionnaires to key stakeholders, including veterans and PACT clinicians, to examine implementation barriers to and facilitators of the intervention.

### Ethical Considerations

This study was approved by the Veterans Affairs (VA) Central Institutional Review Board (CIRB; IRBNet ID 1612635). To obtain participants’ informed consent, a research assistant (RA) will determine whether eligible patients have the capacity to provide informed consent to participate in the study using a screening questionnaire that assesses the 4 elements required for capacity understanding of study procedures, appreciation of what will happen if enrolled, communication of a choice to enroll or not, and demonstration of a rationale for that choice. Recruitment and written informed consent will take place in a location that ensures privacy and convenience for the patient.

### Conceptual Framework of Medication Prescribing in Older Adults

**Figure 1:**
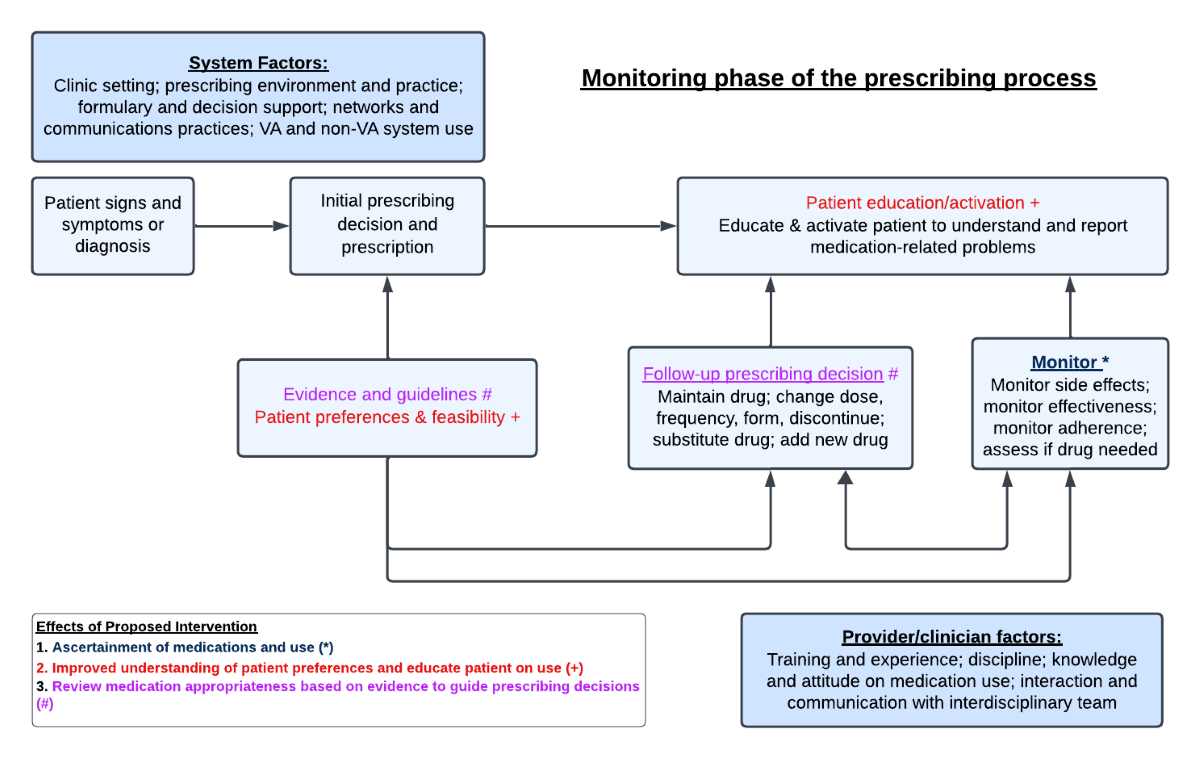
Conceptual framework of prescribing and prescription-monitoring process. Modified from Steinman et al [31]. VA: Veterans Affairs.

Our conceptual framework is informed by the CFIR [34] and Steinman et al [35] on prescribing and prescription monitoring processes. The pilot phase was primarily informed by the Steinman model of the prescribing process adapted to include system- and provider-level factors. The system-level factors include the Veterans Health Administration (VHA) policy on medication management (including non-VA care use), facility-level prescribing environment and practices, and decision support systems. In designing our proposed intervention, we considered the potential effect of home televisits by pharmacists to ascertain the medication regimen and use, identify discrepancies and educate patients on use, and review medication appropriateness, thereby enhancing steps in the monitoring phase of the prescribing process (Figure 1).

### Cluster Randomized Controlled Trial Patient Recruitment

The inclusion criteria include the following: (1) the veteran must be a PACT patient in a Bronx VA or Bedford VA geriatrics or primary care clinic; (2) be 65 years or older; (3) have 2 or more chronic conditions; and (4) have 5 or more medications listed on the VA medication record continuously in the previous 6 month. Patients fulfilling these criteria will be approached for study enrollment through letters of invitation after approval by human subjects’ committees.

### Cluster Assignment by PACT

We will randomize patients by PACT to prevent a team from having participants in both treatment and control groups and to reduce contamination. Prior to initiating enrollment, the project coordinator will assign PACTs to intervention and control groups using lists of computer-generated random numbers in a 1:1 ratio, with separate lists for the Bronx VA and the Bedford VA, in order to maintain balance in both groups within each study site. There are 69 primary care and geriatric PACTs in the Bronx VA and 14 in the Bedford VA, and each PACT will keep the group assignment for the duration of the study.

### Study Flow

After enrollment, veterans who agree to participate in the study will be interviewed by a trained research coordinator using survey instruments that will include data elements listed in Table 1. Veterans will receive the televisit intervention, or not, based on the PACT team they belong to, as discussed earlier. The detailed procedure for control and intervention groups is described later. To observe what occurs at follow-up appointments with the PACT provider, after the first follow-up appointment postintervention, participants will be asked about whether medication use was discussed during the appointment, whether medication reconciliation was performed and how, and what, if any, medication changes were made. Electronic charts will be reviewed for documentation of medication reconciliation and management during the visit. Primary outcomes will be ascertained at 6 months after study enrollment and are described in Table 1, including the subject survey and chart review. The subject survey for outcome assessment will be conducted by a research coordinator trained to take the subject’s medication history, and the chart review will be conducted by a clinician blinded to the study assignment. The subject survey will include confirmation of medications participants are currently taking, including names, dosages, and frequency. Participants will be asked to list medications that they take, including over-the-counter drugs and supplements. A clinician rater blinded to the study assignment will review medication data from the subject survey and chart review to determine medication discrepancies and appropriate use of medications.

**Table 1:** Data elements and instruments.

Data elements		Source	Instruments or measurements
**Outcomes**			
	Primary: PIM^a^ use	Chart review, interview	STOPP^b^ criteria, assessment of medication list by blinded clinical reviewer [14]
	Secondary: medical discrepancies	Chart review, interview	Medication discrepancies (omissions, duplications, additions)
	Medication appropriateness	Chart review, interview	MAI^c^ [36]
	Patient satisfaction, self-efficacy, and adherence	Interview	CAHPS^d^ item pertaining to medication use [37], MUSE^e^ [38]
	Health-related quality of life	Interview	EQ-5D-5L [39]
	ADRs^f^	Chart review, interview	ADRs determination by clinical reviewer
**Baseline covariates**			
	Sociodemographics	Baseline survey, chart review	Gender, age, race, ethnicity, education, income, Medicare, Medicaid
	Chronic illness burden	Patient interview, chart review	Modified RAND index [40]
	Medication list	Chart review, patient interview	Number and type of medications, source (VA^g^, non-VA, over the counter)
	Medication use	Interview, chart review	Number and type of medications, method of administration, presence of refill gap (>90 days)
	Patient’s self-efficacy on medication use	Patient interview	MUSE
	Health literacy	Patient interview	Short Test of Functional Health Literacy in Adults (S-TOFHLA) [41]
	Cognitive function	Patient interview	MoCA^h^ [42]
	Physical function	Patient interview	Katz Activities of Daily Living (ADL) index/Lawton Instrumental Activities of Daily Living (IADL) scale [43,44]
	History of acute care use (hospitalization, emergency department)	Patient interview, chart review	Days prior to enrollment for most recent hospitalization and emergency department visit, number of episodes in the previous year
	Health-related technology use attitude, self-efficacy, and comfort	Patient interview	Self-reported comfort and confidence in using technology [45]

^a^PIM: potentially inappropriate medication.

^b^STOPP: Screening Tool of Older Persons’ Prescriptions.

^c^MAI: Medication Appropriateness Index.

^d^CAHPS: Consumer Assessment of Healthcare Providers and Systems.

^e^MUSE: Medication Understanding Use and Self-Efficacy Scale.

^f^ADR: adverse drug reaction.

^g^MoCA: Montreal Cognitive Assessment.

### Intervention

Patients assigned to the intervention will have a pharmacist televisit appointment made and coordinated. Participants will be asked whether they have a device at home capable of supporting televisits, including home computers with cameras, tablets, smartphones, and a broadband or 4G connection. Participants who do not have appropriate devices will be provided with VA-issued internet-enabled tablets on which to conduct the visits. On the scheduled day of the visit, the research team will coordinate with the participants over the phone to facilitate the initialization of the televisit by the pharmacist. The content of the televisit is described next, in the *Design of the Televisit Intervention* section, and has been adapted through the pilot phase with direct observation of the televisit at home. Subsequent to the televisit, the pharmacist will document the visit content in the electronic medical record (EMR) to note discrepancies noted during the televisit, a review of medications with START/STOPP criteria, and recommendations based on the criteria-based review for PCP review and concurrence for adjustments of medications. Recommendations will be communicated to the PCP electronically through secure email or an electronic note or over the phone or through a face-to-face discussion if preferred by the PCP. Adjustments in the medication regimen will be noted at 7 days in the EMR after recommendations are made. If recommendations are not adopted, the pharmacist will communicate with the PCP electronically to request reasons for not adopting recommendations. Participants will then continue follow-up primary care with their PCP.

### Design of the Televisit Intervention

We plan to use the VA video connect (VVC) capability introduced in fiscal year 2018 to conduct televisits between clinical pharmacists located at the VA medical centers and participants at home. An appointment will be scheduled with each participant for time to conduct the home televisit. The devices, EX90s or web cameras, used at the clinician side will be located at the Bronx VA or the Bedford VA and at the participants’ side will be their choice of computer, tablet, or smartphone with an internet connection and webcam capability or a VA-provided tablet if they do not have a device or home internet capabilities. Because the experience of the televisit may differ with different participant devices, various devices were tested in phase I of the study, and their strengths and pitfalls and modes of connecting were identified.

After establishing a video connection, the pharmacist will conduct home-based medication reconciliation by asking the participants to show and explain the use of each of their medications. The pharmacist will also include a brief medication-focused functional assessment, including asking the participants to read aloud and interpret 1 or more of their prescription bottles and to demonstrate the ability to open the bottles. Before ending the visit, the pharmacist will educate the participants regarding possible side effects or interactions between their medications. The pharmacist will also solicit and answer any questions the participants have about the medications. The pharmacist will note the medications used and compare them against the list of medications on each participant’s EMR to note discrepancies and then generate a note in the EMR to notify the primary care team of the review and information obtained. If the pharmacist finds that patients are taking medications differently, they will clarify the correct usage of the medications.

#### Pharmacist Review of Medications

After the televisit is completed, the pharmacist will conduct a medication review using the START/STOPP criteria to review the indication of each medication (see Table 2). They will also review whether there are medications prescribed beyond the recommended duration of each medication, where the treatment duration is well defined. The pharmacist will use evidence-based guidelines to inform follow-up prescribing decisions (maintaining, adjusting the dose, or stopping the medication); see Figure 1. Medications noted to be inappropriate or those that do not have an indication will be flagged for consideration of deprescribing. Duplication of drug class prescriptions will also be noted. Other disease-specific criteria will be reviewed based on the criteria [13-15]. Recommendations generated from the medication review based on START/STOPP criteria will be communicated to the PCP for consideration of modification of the medication regimen. Final decisions on medication modification will be made by primary care clinicians in consultation with their patients as it usually would be in a clinical setting.

**Table 2:** Scope of pharmacist-led intervention televisit, laying out the steps, content, and approximate duration of each step.

Step	Content and sample questions	Approximate duration (60-75 minutes total)
Setup	Establish a connection, preparation for the visit.	5-10 minutes
Introduction	Identify individuals in the visit and discuss the purpose of the visit.	5 minutes
Identification of the medication regimen and visualization of medications	What medicines do you currently take? How about over-the-counter medicines? How about vitamins and supplements? Where do you keep your medications? Can you show me?	15 minutes
Description and visualization of how to take medications	What do you take this medicine for? When do you take this medicine? Can you show me how much you take each time?	15-20 minutes
Clarify medication instructions	If medications are taken incorrectly, ask why. Educate and use the teach-back method to confirm understanding.	15 minutes
Questions and closing	Answer the patient’s questions, if any. Discuss the next steps of review and communication with the PCP^a^.	5-10 minutes

^a^PCP: primary care provider.

### Control Arm (Usual Care)

After baseline MoCA and technology comfort assessment, medication reconciliation and management will be conducted by the control primary care teams in the manner they usually do. PACTs are guided by the VHA Directive on Medication reconciliation [46], which includes in-clinic assessment of medication information provided by patients via lists, recall or actual medication reviews, comparing information obtained to the VA EMR to note discrepancies, and educating the patient on updated medications. PACT PCPs may also request assessment of medication regimens by pharmacists embedded in their clinic based on clinical decisions regarding the needs of the individual patient and the availability of such services. The prescription of a new medication may trigger a review by a VA pharmacist guided by EMR-based interaction alerts. We considered using an active control group with pharmacist review (in-clinic or chart review); however, the intervention with pharmacist-led home televisits informing medication review represents a bundled intervention, and the study will examine the effectiveness of the intervention as an enhancement of care to what currently occurs (usual care). This design involving participant interviews and chart reviews will also allow us to characterize current usual medication management for older adults in PACTs.

### Outcomes

#### Primary Outcome

PIM use, which is the primary outcome, will be assessed by the blinded clinical reviewer based on the medication data obtained from home visit surveys using the STOPP criteria [14]. Proportions of participants with PIMs in the intervention group will be compared with proportions of participants with PIMs in the control group.

#### Secondary Outcomes

Medication lists obtained through subject surveys will be compared against the EMRs by a clinician to establish discrepancies in medications. Discrepancies will be characterized as “no potential harm,” “monitoring or intervention potentially to preclude harm,” or “potential harm,” similar to prior approaches to assess potential clinical impacts [32]. In addition, the clinical reviewer will assess the medications using the validated Medication Appropriateness Index (MAI) [36]. Each medication will receive a score based on the 10-item tool to determine its appropriateness. Patient satisfaction will be ascertained using an item pertaining to medication use from Consumer Assessment of Healthcare Providers and Systems (CAHPS) version 3.0 [37], a 7-item, 5-point Likert scale on medication management by pharmacists adapted from a validated instrument [33,47]; self-efficacy on medication use will be assessed using the Medication Understanding Use and Self-Efficacy Scale (MUSE) to assess change from before and after the intervention [38]. Health-related quality of life will be assessed using the EQ-5D-5L instrument, a brief validated instrument with good test-retest reliability [39,48].

### Implementation Factors

Postintervention questionnaires will be administered to key stakeholders, including veteran participants and PACT clinicians, to assess the potential for future implementation of the intervention. This will allow us to examine implementation barriers and facilitators. An invitation to complete the postintervention questionnaires will be emailed to PACT providers in the intervention arm. To ensure we understand PCP decisions on pharmacist recommendations, we will track PCP adoption of recommendations provided by pharmacist reviews and examine PCP reasons for adoption or nonadoption of recommendations, as well as provider factors that can influence those decisions. Patient and clinician interviews and questionnaires will identify perceived barriers and facilitators on a Likert scale. The knowledge and attitude on prescribing for older adults will also be obtained using validated Likert questions, although the attitude and behavior in clinician communication with pharmacists will be gathered using the Home Medicines Review Inventory (HMRI).

## Results

**Figure 2:**
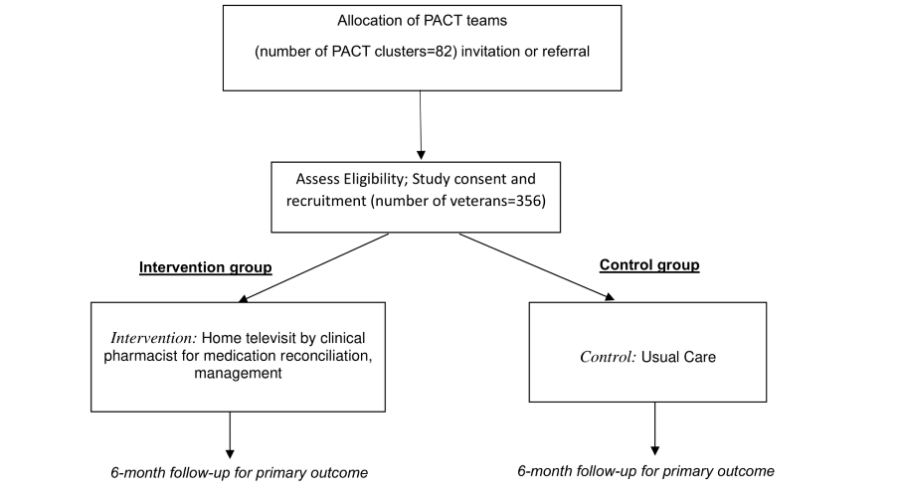
Study design showing specific steps included in intervention and control groups. PACT: patient-aligned care team.

The study flow is summarized in Figure 2. The pilot phase of the study was completed December 2021 with 20 veterans and was primarily informed by the Steinman model of the prescribing process adapted to include system- and provider-level factors. This project has been peer-reviewed and selected for support by the VA Health Services Research Service. The last date of enrollment was August 6, 2021. We anticipate the completion of the ongoing trial in spring 2025. The first results are expected to be submitted for publication in 2025.

## Discussion

### Summary

The complex medication regimens of many older adults contribute to increased risk for drug interactions, ADRs, and other poor health outcomes. A number of studies have found that the use of PIMs is associated with adverse outcomes, such as falls, acute care use, and other negative outcomes [49,50]. Improving medication prescribing using START/STOPP criteria has been found to reduce adverse effects and improve patient outcomes in a number of studies, although mostly in institution-based settings (acute care hospitalization, nursing homes) [50]. Pharmacist-led interventions have been demonstrated to have beneficial effects [51,52] but often do not reach the majority of those who would most benefit from them. Leveraging the advances of telemedicine, clinicians can provide medication management services to older veterans at home. The televisit intervention with pharmacists in medication management has the potential to bridge the current gaps in older adult care and provide a scalable solution to improve medication use in older adults.

The study’s pilot phase refined the procedure for pharmacists to conduct home televisits. It is possible that veterans will consider televisits to home cumbersome or invasive and that VA primary care staff will consider tailoring medications for older adults an additional task burden that competes with other mandated tasks. Our prior experiences of home televisits suggest that they are well accepted, and our hybrid effectiveness implementation study will enable us to identify potential implementation challenges and thus can inform future implementation. To ensure we understand PCP decisions on pharmacist recommendations, we will track PCP adoption of recommendations provided by pharmacist reviews to identify PCP reasons for adoption or nonadoption of recommendations, as well as provider factors that can influence those decisions.

The older adult population has increased the use of technology, allowing the VA to use technological advances for VA televisits, overcoming previous barriers, and we expect future changes in technology will further facilitate use. Our study design will allow us to observe older adults using technology at home to enhance our understanding of the barriers to use, and qualitative interviews with clinicians and patients will further identify factors to guide future implementation. We have elected to use 2 separate sites in the RCT phase in order to enhance our understanding of contextual issues and external validity.

### Strengths and Limitations

This study design includes both a pilot phase trial and a hybrid effectiveness implementation trial, which ensures that we would observe and be able to address challenges prior to the RCT, although the results of the hybrid implementation effectiveness trial would allow us to determine its effectiveness, while preparing us to spread the intervention, if effective, in the health system. We aim to limit crossover contamination for providers with cluster randomization. Lastly, the standardized intervention and structured pharmacist protocol will ensure consistency and reliability.

The study design also has a few limitations. One is that the study will be conducted in urban and suburban settings, which may limit generalizability Another limitation is that the pilot phase was conducted during the COVID-19 pandemic, which introduced unique challenges in recruitment due to factors such as travel restrictions and increased risk for participants; however, the study team tackled those challenges and completed the pilot phase of the study despite limitations introduced by COVID-19. This study will contribute toward the literature on pharmacist-led medication management through home televisit procedures, as well as interventions aimed at improving medication use in older veterans.

### Conclusion

This study design will demonstrate how medication management will be beneficial through televisits. The implementation effectiveness trial will show how it can improve the use of medications and interventions related to the potential impact on older adults with multiple chronic conditions and polypharmacy. Clinicians can offer older veterans medication management services at home by using telemedicine’s advancements. The use of pharmacists in televisit medication management interventions has the potential to close existing gaps in older adult care and offer a scalable way to enhance older adults’ medication use.

## Appendix

Multimedia Appendix 1 Peer review from the HSR-6 Post-acute and Long-term Care - Department of Veterans Affairs Office of Research and Development - Health Services Research and Development Service (USA).

## Abbreviations

ADR: adverse drug reaction
CAHPS: Consumer Assessment of Healthcare Providers and Systems
CFIR: Consolidated Framework for Implementation Research
EMR: electronic medical record
MAI: Medication Appropriateness Index
MoCA: Montreal Cognitive Assessment
MUSE: Medication Understanding Use and Self-Efficacy Scale
PACT: patient-aligned care team
PCP: primary care provider
PIM: potentially inappropriate medication
RCT: randomized controlled trial
START: Screening Tool to Alert to Right Treatment
STOPP: Screening Tool of Older Persons’ Prescriptions
VA: Veterans Affairs
VHA: Veterans Health Administration

## Data Availability

No data sets will be generated or analyzed during this study.

## References

[1] Conduct brown bag medicine reviews: tool 8. Agency for Healthcare Research and Quality.

[2] Ortman J, Velkoff V, Hogan H The aging nation: the older population in the United States. U.S. Census Bureau.

[3] Ward BW, Schiller JS (2013). Prevalence of multiple chronic conditions among US adults: estimates from the National Health Interview Survey, 2010. Prev Chronic Dis.

[4] Hung WW, Ross JS, Boockvar KS, Siu AL (2011). Recent trends in chronic disease, impairment and disability among older adults in the United States. BMC Geriatr.

[5] Boyd CM, Darer J, Boult C, Fried LP, Boult L, Wu AW (2005). Clinical practice guidelines and quality of care for older patients with multiple comorbid diseases: implications for pay for performance. JAMA.

[6] Tinetti ME, Bogardus ST, Agostini JV (2004). Potential pitfalls of disease-specific guidelines for patients with multiple conditions. N Engl J Med.

[7] (2018). Patterns of medication use in the United States, 2006. Slone Epidemiology Center at Boston University.

[8] Steinman MA, Hanlon JT (2010). Managing medications in clinically complex elders: "there's got to be a happy medium". JAMA.

[9] Steinman MA, Landefeld CS, Rosenthal GE, Berthenthal D, Sen S, Kaboli PJ (2006). Polypharmacy and prescribing quality in older people. J Am Geriatr Soc.

[10] Vik SA, Maxwell CJ, Hogan DB (2004). Measurement, correlates, and health outcomes of medication adherence among seniors. Ann Pharmacother.

[11] Mather GG, Levy RH (1996). Pharmacokinetics of polypharmacy: prediction of drug interactions. Epilepsy Res Suppl.

[12] Gurwitz JH, Field TS, Harrold LR, Rothschild J, Debellis K, Seger AC, Cadoret C, Fish LS, Garber L, Kelleher M, Bates DW (2003). Incidence and preventability of adverse drug events among older persons in the ambulatory setting. JAMA.

[13] Bjerre LM, Ramsay T, Cahir C, Ryan C, Halil R, Farrell B, Thavorn K, Catley C, Hawken S, Gillespie U, Manuel DG (2015). Assessing potentially inappropriate prescribing (PIP) and predicting patient outcomes in Ontario's older population: a population-based cohort study applying subsets of the STOPP/START and Beers' criteria in large health administrative databases. BMJ Open.

[14] Blanco-Reina E, García-Merino MR, Ocaña-Riola R, Aguilar-Cano L, Valdellós J, Bellido-Estévez I, Ariza-Zafra G (2016). Assessing potentially inappropriate prescribing in community-dwelling older patients using the updated version of STOPP-START criteria: a comparison of profiles and prevalences with respect to the original version. PLoS One.

[15] Wallace E, McDowell R, Bennett K, Fahey T, Smith SM (2017). Impact of potentially inappropriate prescribing on adverse drug events, health related quality of life and emergency hospital attendance in older people attending general practice: a prospective cohort study. J Gerontol A Biol Sci Med Sci.

[16] American Geriatrics Society (2015). 2015 Updated Beers criteria for potentially inappropriate medication use in older adults. J Am Geriatr Soc.

[17] Levy HB (2017). Polypharmacy reduction strategies: tips on incorporating American Geriatrics Society Beers and screening tool of older people's prescriptions criteria. Clin Geriatr Med.

[18] Smith S (2017). Telemedicine: an important tool for veterans health. United States Department of Veterans Affairs.

[19] Agha Z, Schapira RM, Laud PW, McNutt G, Roter DL (2009). Patient satisfaction with physician-patient communication during telemedicine. Telemed J E Health.

[20] Mair F, Whitten P (2000). Systematic review of studies of patient satisfaction with telemedicine. BMJ.

[21] van den Berg N, Schumann M, Kraft K, Hoffmann W (2012). Telemedicine and telecare for older patients--a systematic review. Maturitas.

[22] Walker CL, Kopp M, Binford RM, Bowers CJ (2017). Home telehealth interventions for older adults with diabetes. Home Healthc Now.

[23] Littauer SL, Dixon DL, Mishra VK, Sisson EM, Salgado TM (2017). Pharmacists providing care in the outpatient setting through telemedicine models: a narrative review. Pharm Pract (Granada).

[24] Niznik JD, He H, Kane-Gill SL (2018). Impact of clinical pharmacist services delivered via telemedicine in the outpatient or ambulatory care setting: a systematic review. Res Social Adm Pharm.

[25] Moo LR, Jafri Z, Morin PJ (2014). Home-based video telehealth for veterans with dementia. Fed Pract.

[26] Singh L, Accursi M, Korch Black K (2015). Implementation and outcomes of a pharmacist-managed clinical video telehealth anticoagulation clinic. Am J Health Syst Pharm.

[27] Curran GM, Bauer M, Mittman B, Pyne JM, Stetler C (2012). Effectiveness-implementation hybrid designs: combining elements of clinical effectiveness and implementation research to enhance public health impact. Med Care.

[28] Bauer MS, Damschroder L, Hagedorn H, Smith J, Kilbourne AM (2015). An introduction to implementation science for the non-specialist. BMC Psychol.

[29] (2018). CY 2019 medication therapy management program guidance and submission instructions. Center for Medicare Services.

[30] Hajjar ER, Hanlon JT, Artz MB, Lindblad CI, Pieper CF, Sloane RJ, Ruby CM, Schmader KE (2003). Adverse drug reaction risk factors in older outpatients. Am J Geriatr Pharmacother.

[31] Hawley CE, Wagner C, Venegas MD, Genovese N, Triantafylidis LK, McCullough MB, Beizer JL, Hung WW, Moo LR (2024). Connecting the disconnected: leveraging an in-home team member for video visits for older adults. J Am Geriatr Soc.

[32] Quélennec B, Beretz L, Paya D, Blicklé JF, Gourieux B, Andrès E, Michel B (2013). Potential clinical impact of medication discrepancies at hospital admission. Eur J Intern Med.

[33] Flanagan P, Kainth S, Nissen L (2013). Satisfaction survey for a medication management program: satisfaction guaranteed?. Can J Hosp Pharm.

[34] Damschroder LJ, Aron DC, Keith RE, Kirsh SR, Alexander JA, Lowery JC (2009). Fostering implementation of health services research findings into practice: a consolidated framework for advancing implementation science. Implement Sci.

[35] Steinman MA, Handler SM, Gurwitz JH, Schiff GD, Covinsky KE (2011). Beyond the prescription: medication monitoring and adverse drug events in older adults. J Am Geriatr Soc.

[36] Hanlon JT, Schmader KE, Samsa GP, Weinberger M, Uttech KM, Lewis IK, Cohen HJ, Feussner JR (1992). A method for assessing drug therapy appropriateness. J Clin Epidemiol.

[37] Agency for Healthcare Research and Quality.

[38] Cameron KA, Ross EL, Clayman ML, Bergeron AR, Federman AD, Bailey SC, Davis TC, Wolf MS (2010). Measuring patients' self-efficacy in understanding and using prescription medication. Patient Educ Couns.

[39] Rabin R, de Charro F (2001). EQ-5D: a measure of health status from the EuroQol Group. Ann Med.

[40] Keeler EB (1990). Changes in sickness at admission following the introduction of the prospective payment system. JAMA.

[41] Baker DW, Williams MV, Parker RM, Gazmararian JA, Nurss J (1999). Development of a brief test to measure functional health literacy. Patient Educ Couns.

[42] Nasreddine ZS, Phillips NA, Bédirian V, Charbonneau S, Whitehead V, Collin I, Cummings JL, Chertkow H (2005). The Montreal Cognitive Assessment, MoCA: a brief screening tool for mild cognitive impairment. J Am Geriatr Soc.

[43] Katz S, Akpom CA (1976). 12. Index of ADL. Med Care.

[44] Lawton MP, Brody EM (1969). Assessment of older people: self-maintaining and instrumental activities of daily living. Gerontologist.

[45] Zulman DM, Piette JD, Jenchura EC, Asch SM, Rosland A (2013). Facilitating out-of-home caregiving through health information technology: survey of informal caregivers' current practices, interests, and perceived barriers. J Med Internet Res.

[46] United States Department of Veterans Affairs (2011). VHA Directive 2011-012. Medication Reconciliation.

[47] Ramalho de Oliveira D, Brummel AR, Miller DB (2010). Medication therapy management: 10 years of experience in a large integrated health care system. J Manag Care Pharm.

[48] van Agt HM, Essink-Bot M, Krabbe PF, Bonsel GJ (1994). Test-retest reliability of health state valuations collected with the EuroQol questionnaire. Soc Sci Med.

[49] Dalleur O, Spinewine A, Henrard S, Losseau C, Speybroeck N, Boland B (2012). Inappropriate prescribing and related hospital admissions in frail older persons according to the STOPP and START criteria. Drugs Aging.

[50] Hill-Taylor B, Walsh KA, Stewart S, Hayden J, Byrne S, Sketris IS (2016). Effectiveness of the STOPP/START (Screening Tool of Older Persons' potentially inappropriate Prescriptions/Screening Tool to Alert doctors to the Right Treatment) criteria: systematic review and meta-analysis of randomized controlled studies. J Clin Pharm Ther.

[51] Gillespie U, Alassaad A, Hammarlund-Udenaes M, Mörlin C, Henrohn D, Bertilsson M, Melhus H (2013). Effects of pharmacists' interventions on appropriateness of prescribing and evaluation of the instruments' (MAI, STOPP and STARTs') ability to predict hospitalization--analyses from a randomized controlled trial. PLoS One.

[52] Gillespie U, Alassaad A, Henrohn D, Garmo H, Hammarlund-Udenaes M, Toss H, Kettis-Lindblad ?, Melhus H, Mörlin C (2009). A comprehensive pharmacist intervention to reduce morbidity in patients 80 years or older: a randomized controlled trial. Arch Intern Med.

