# Supplementary material for: Effect of Medication Management at Home via Pharmacist-Led Home Televisits: Protocol for a Cluster Randomized Controlled Trial
Source: JMIR Res Protoc. 2025 Feb 5;14:e65141. doi: 10.2196/65141 (PMC11840363; doi:10.2196/65141)
Supplement: Multimedia Appendix 1 [file resprot_v14i1e65141_app1.pdf]

KEY SUMMARY POINTS:

1. Important research for mission of VA that incorporates pharmacy led intervention.
2. Highly qualified team with expertise in the important areas.
3. Some concern that the study may be incremental, although focus on actual use by patient is innovative.
4. Proposal responds well to prior critiques, although justification in Aim 2 for two sites is not provided (especially for explaining statistical power).

DESCRIPTION (provided by applicant):

Older adults are more likely to suffer from multiple chronic conditions, to be prescribed multiple medications, and are more susceptible to adverse effects of medications. In addition, older adults often use over-the-counter medications and supplements, further complicating their medication regimen. Complex medication regimens are potentially harmful to older adults due to potential drug interactions, potentially inappropriate prescribing or over-the-counter drug use, and medication non-adherence that may lead to poor control of chronic disease. Interventions aimed at reducing medication discrepancy in the ambulatory clinic setting, such as the review of written medication lists, and implementation of “brown bag” reconciliation (asking patients to bring in all medication bottles for review in the clinic) continues to be challenging and have limited success. Clinical pharmacist led interventions to improve appropriate medication use in older adults, including the application of the START/STOPP criteria, have demonstrated effectiveness in reducing adverse drug events. With the increased capability of VA telemedicine to reach Veteran in their homes, delivering medication management via televisit by clinical pharmacists has the potential to yield similar benefits for a larger number of older Veterans. Telemedicine is an increasingly vital component within VHA to increase access and improve quality of care. By extending care beyond brick-and-mortar clinics, telemedicine increases the reach of care teams and is more convenient for patients, resulting in improved patient satisfaction. Using the capability of telemedicine to reach patients; homes, we propose to examine the effect of medication management by clinical pharmacists via home video televisits, as home video visits have the potential to provide direct visualization of medications in older adults; homes, thereby reducing medication discrepancy and increasing medication adherence. Pharmacist management for older adult medication regimen may also improve appropriate medication use in older adults through direct pharmacist-patient interview and education. In support of this application, preliminary data from our team of investigators demonstrate acceptability of video televisits by older adults, that there is good uptake by patients and VA providers, and that video televisits into the home are feasible. In this study, we aim to develop a protocol for pharmacy home televisits for medication management in older adults who have multiple chronic conditions and are on multiple medications. We will then conduct a randomized trial with hybrid effectiveness Type I design to examine the effect of these televisits on appropriate medication use, medication discrepancies, adherence and adverse drug events and observe and gather information on implementation. We anticipate that a pharmacist led medication management home televisit intervention will lead to reduction in potentially inappropriate use of medication, reduction in medication discrepancies, increased medication adherence and reduced adverse drug events in older adults compared to older adults receiving usual care. We will also examine the barriers and facilitators in implementing the intervention so that the study findings may inform future implementation.

Review 1. The proposed outcomes of this work will lead allow for further work to scale telehealth to reduce inappropriate use of medication, reduce medication discrepancies, increase medication adherence and reduce adverse drug events in older adults compared to older adults receiving usual care.

1. Telemedicine is an increasingly vital component within Veterans Health Administration (VHA) to increase access, improve quality of care and increase scalability with advantages compared to home visits as telemedicine has the potential to provide direct visualization of medication in the Veterans home.

2. Pharmacist lead interventions have proven beneficial beyond physician managed medication interventions.

3. Methodologically strong with appropriate Type 1 hybrid design, along with qualitative and quantitative research.

4. One of the most significant strengths of this work is the highly qualified team with significant experience and long-standing collaboration in implementing telemedicine (Dr. Huang and Dr. Moo) and medication reconciliation and management in older adults (Dr. Boockvar) and a geriatrics pharmacist.

5. Significant research has been conducted to date regarding telehealth and pharmacist lead medication across the VA. Uncertain of the significance of this proposal.

6. Control groups should include participants will receive usual care in which medication reconciliation and review is conducted by clinical pharmacists in clinics and not by primary care teams.

7. It is common for follow up recommendations to be low. It is not clear how will the pharmacist will communicate with PCP “electronically” to request reasons for not adopting recommendations. Clarification on how these data will be recorded, and measured, and reported is needed.

8. Significant time, effort and cost for a 20 Veteran cohort evaluation matched with “usual care.”

Review 2. This is a scientifically and methodologically sound and technologically feasible project aimed at improving assessment of medication management by community-residing older Veterans.

1. Ample scientific and clinical justification for the study.

2. Prior investigations by this team provide a firm foundation for this project.

3. Establishment of ground truth via concurrent in-home and remote observation for Aim 1.

4. Qualified team with the methodological, technical, and domain expertise needed to carry out the proposed research.

5. Thorough plan for medication management assessment, provision of indicated recommendations to providers, and timely follow-up to determine disposition of those recommendations.

6. No key weaknesses are noted.

Review 3. This is an innovative proposal submitted by an excellent team. The application would be strengthened if it could present the need to have a 2 - site study.

1. Strong study team.

2. Interesting study question.

3. Addressed most of the previous reviewer concerns.

4. The justification for the 2-site approach is not well described
